# Efficacy and safety of lurasidone in acutely psychotic patients with schizophrenia: A 6‐week, randomized, double‐blind, placebo‐controlled study

**DOI:** 10.1111/pcn.13221

**Published:** 2021-05-21

**Authors:** Masaomi Iyo, Jun Ishigooka, Masatoshi Nakamura, Reiko Sakaguchi, Keisuke Okamoto, Yongcai Mao, Joyce Tsai, Alison Fitzgerald, Tadashi Nosaka, Teruhiko Higuchi

**Affiliations:** ^1^ Department of Psychiatry Graduate School of Medicine, Chiba University Chiba Japan; ^2^ Institute of CNS Pharmacology Tokyo Japan; ^3^ Department of Data Science, Drug Development Division Sumitomo Dainippon Pharma Co., Ltd. Tokyo Japan; ^4^ Department of Clinical Research Drug Development Division, Sumitomo Dainippon Pharma Co., Ltd. Tokyo Japan; ^5^ Department of Clinical Operation Drug Development Division, Sumitomo Dainippon Pharma Co., Ltd. Tokyo Japan; ^6^ Division of Data Science Sunovion Pharmaceuticals Inc. New Jersey USA; ^7^ Division of Clinical Research Sunovion Pharmaceuticals Inc. New Jersey USA; ^8^ Division of Clinical Operations Sunovion Pharmaceuticals Inc. New Jersey USA; ^9^ Medical Affairs Sumitomo Dainippon Pharma Co., Ltd. Tokyo Japan; ^10^ Japan Depression Center Tokyo Japan; ^11^ National Center of Neurology and Psychiatry Tokyo Japan

**Keywords:** antipsychotic agents, clinical trial, efficacy, lurasidone, schizophrenia

## Abstract

**Aim:**

The aim of this study was to evaluate the efficacy of lurasidone in acute schizophrenia in Japan and other countries.

**Methods:**

Subjects (aged 18–74 years) diagnosed with schizophrenia were randomized to lurasidone 40 mg/day or placebo. The primary efficacy endpoint was change from baseline on the Positive and Negative Syndrome Scale (PANSS) total score at Week 6. Secondary efficacy assessments included the Clinical Global Impression‐Severity Scale (CGI‐S). Safety endpoints included adverse events, and laboratory and electrocardiogram parameters.

**Results:**

A total of 483 subjects were randomized to lurasidone or placebo; 107 subjects were from Japan. Mean changes from baseline at Week 6 endpoint in PANSS total scores were −19.3 in the lurasidone group and −12.7 in the placebo group (treatment difference: *P* < 0.001, effect size = 0.41). Changes from baseline for Week 6 CGI‐S scores were −1.0 for lurasidone and −0.7 for placebo (treatment difference: *P* < 0.001, effect size = 0.41). All‐cause discontinuation during the 6‐week, double‐blind period was 19.4% for lurasidone and 25.4% for placebo, and discontinuation rates due to adverse event were 5.7% for lurasidone and 6.4% for placebo. The following common treatment‐emergent adverse events occurred in more than 2% on lurasidone and at a rate at least twice that of the placebo group: akathisia (4.0%), dizziness (2.8%), somnolence (2.8%), abdominal discomfort (2.0%) and asthenia (2.0%). No significant changes in bodyweight or metabolic parameters were observed.

**Conclusion:**

Lurasidone 40 mg once daily dosing demonstrated efficacy in a patient population with acute schizophrenia, including subjects from Japan, and was generally safe and well‐tolerated.

Globally, schizophrenia is estimated to affect at least 20 million people.^1^ Impairments in functioning are extensive with schizophrenia, ranking it among the 20 leading causes of disability.^2^, ^3^ Reduced lifespan is also apparent among people with schizophrenia, primarily related to increased rates of suicide and cardiovascular disease.^4^, ^5^, ^6^

Lurasidone is a benzisothiazole, second‐generation antipsychotic drug marketed in the USA, Canada, the European Union, Switzerland, Australia, and Brazil for the treatment of schizophrenia in recommended doses between 40 and 160 mg/day. This novel compound possesses potent antagonist affinity for dopamine D_2_ and serotonin 5‐HT_2A_ and 5‐HT_7_ receptors, moderate antagonist affinity at α_2A_ and α_2C_ adrenergic receptors, and partial agonist affinity at 5‐HT_1A_ receptors.^7^ Unlike some other second‐generation antipsychotics, lurasidone has either no or minimal affinity for the 5‐HT_2C_ receptors, histamine H_1_ receptors, and muscarinic M_1_ receptors that are related to weight gain, metabolic syndrome, or sedation.^7^

The efficacy, safety, and tolerability of lurasidone in the treatment of schizophrenia has been demonstrated in a number of studies conducted in the USA, Europe, Asia, and South America. A meta‐analysis of eight placebo‐controlled, short‐term studies involving 2373 patients with schizophrenia found that lurasidone at doses up to 160 mg/day was superior to placebo with regard to change in total psychopathology, positive symptoms, negative symptoms, and general psychopathology.^8^ Lurasidone has not been found to be significantly associated with metabolic dysfunction as measured by changes in triglycerides, total cholesterol, high‐density lipoprotein cholesterol, low‐density lipoprotein cholesterol, fasting glucose, HbA1c, fast insulin, or prolactin level compared to placebo.^8^

None of the eight placebo‐controlled trials included in the meta‐analysis involved individuals with schizophrenia from Japan.^8^ Two recent clinical trials have been conducted that included patients with schizophrenia from Japan. In the first trial, no significant endpoint differences on the primary outcome were found for either dose of lurasidone (40 mg/day or 80 mg/day) or risperidone versus placebo.^9^ However, the selection criteria of this trial were different from previous trials conducted in USA that had reported positive results for lurasidone.^10^, ^11^, ^12^ As with the US trials, the current study increased the severity criteria required for entry from a Positive and Negative Syndrome Scale (PANSS)^13^ total score ≥70 to a PANSS total score ≥80; and it required patients to be experiencing an acute exacerbation of mainly positive symptoms (e.g., exacerbation of delusion or hallucination; thought disorder). In the second trial, neither lurasidone 40 mg/day nor 80 mg/day significantly separated from placebo on the primary outcome in a modified intent‐to‐treat analysis. A full intent‐to‐treat (ITT) analysis conducted as a secondary analysis did find that lurasidone 40 mg and 80 mg were superior to placebo.^14^ Although the inclusion/exclusion criteria of this study were generally similar to those of the previous US trials, the study also included partially treatment‐resistant patients and those with primarily negative symptoms; and these sample characteristics may have reduced the degree of study sensitivity.

The inconsistent results of the Japan studies raised the question of the generalizability of the lurasidone acute schizophrenia efficacy findings across various populations, including Japanese. Adding to this question were previous studies that have found ethnic/nationality differences in response to some antipsychotics.^15^, ^16^, ^17^ Further research on the generalizability of the effects of lurasidone in the treatment of schizophrenia was therefore warranted. The primary objective of the current study was to conduct a Phase 3 evaluation of the efficacy and safety of lurasidone in a geographically diverse sample of subjects, including some from Japan, with acute exacerbation of schizophrenia and the presence of positive symptoms.

## Methods

### Study design and participants

This was a multicenter, randomized, double‐blind, placebo‐controlled, parallel‐group study designed to evaluate the efficacy and safety of lurasidone 40 mg/day administered over a 6‐week period in patients with acute schizophrenia (clinical trial registration, EudraCT number: 2016–000060‐42; study initiated May 2016 and completed November 2018). The study consisted of a screening/washout phase (up to 21 days, including a 3‐ to 7‐day single‐blind washout phase) followed by a double‐blind treatment phase (6 weeks) and a follow‐up visit (7 days following last dose of study drug). Hospitalization was required during the single‐blind washout and from baseline to Week 2 (day 15 ± 2).

This study was conducted in accordance with the International Conference on Harmonization Guideline for Good Clinical Practice and the Declaration of Helsinki at 73 clinical sites in five countries (Japan, Ukraine, Russia, Romania, and Poland). The protocol was approved by the Ethics Committee at each participating center. Written informed consent was obtained from each patient following a detailed explanation of study procedures. This RCT manuscript was written according to the Consolidated Standards of Reporting Trials 2010 guideline.

Patients (aged 18–74 years) were diagnosed with schizophrenia according to a clinical interview using the Mini‐International Neuropsychiatric Interview (MINI)^18^ 6.0.0 with Diagnostic and Statistical Manual of Mental Disorder, 4th Edition, Text Revision (DSM‐IV‐TR)^19^ criteria. To be included in the study, patients also had to meet the following key criteria: a PANSS^13^ total score ≥80; a PANSS item score ≥4 (moderate) on two or more of the following PANSS items: delusions, conceptual disorganization, hallucinations, suspiciousness, or unusual thought content at both screening and baseline; a score of 4 (moderately ill) or higher on the Clinical Global Impressions‐Severity of Illness (CGI‐S)^20^ at screening and baseline; an acute exacerbation of mainly positive symptoms (e.g., exacerbation of delusion or hallucination; thought disorder) for no longer than 2 months prior to the screening visit and marked deterioration of function from baseline (by history); and able to be hospitalized from Visit 2 (washout) through Visit 5 (Week 2) assessments. Key exclusion criteria were: continuous hospitalization for >3 months (90 days) immediately prior to screening; continuous hospitalization for >14 days for acute exacerbation of psychotic symptoms immediately prior to screening in patients who had been treated continuously with adequate doses of one or more antipsychotic agents for ≥4 weeks immediately prior to screening; a decrease of ≥20% in the PANSS total score between screening and baseline visits; PANSS total score below 80 at baseline; patient is considered to be at imminent risk of suicide or injury to self or others; history of treatment with clozapine for refractory psychosis or treatment‐resistant schizophrenia; receiving a total dose of antipsychotic medication equivalent to ≥12.0 mg/day of haloperidol at the screening visit; received any depot antipsychotic drugs (sustained‐release formulation) more recently than the minimum required washout prior to screening; and received fluoxetine within 1 month of screening.

### Randomization and masking

Randomization was implemented (1:1 ratio of drug to placebo) using an Interactive Voice/Web Response System (IXRS) performed at baseline. A unique subject number was assigned by the IXRS when a subject entered the screening period. The unique subject number allocated a patient to a particular treatment group and identified the subject for data collection purposes. Patients, investigators, and all research staff remained blinded to the identity of the treatment from the time of randomization until database lock and unblinding. Study drugs were all identical in packaging, labeling, schedule of administration, and appearance.

### Drug administration and concomitant medications

Study drug consisted of tablets containing either lurasidone 40 mg/day or placebo and was administered orally, once daily in the evening, with food or within 30 min after eating.

When a patient was an inpatient, the hospital pharmacist ensured daily compliance with the dosing regimen. When/if a patient was an outpatient, compliance was monitored closely at each visit. Patients were instructed to bring all unused study drugs with them to each visit. Compliance was assessed by counting tablets and dividing the actual number of doses taken (per tablet count) by the number of doses the subject should have taken within a visit period and multiplying by 100. All patients were reminded of the importance of strict compliance with taking the study drug as directed, with food, for the effectiveness of treatment and for the successful outcome of the study.

Patients were required to discontinue prohibited medications, including antipsychotics, antidepressants, mood stabilizers, and other psychotropics. Potent inducers or inhibitors of the CYP3A4 enzyme system were prohibited during all phases of this study. Biperiden, trihexyphenidyl, diphenhydramine, or promethazine were also permitted if benztropine was not available for the management of treatment‐emergent movement disorders, or if a subject had an inadequate response or intolerability to benztropine treatment. Treatment with propranolol (≤120 mg/day) was permitted as needed for akathisia. Concomitant use of lorazepam, zolpidem, temazepam, brotizolam, triazolam, lormetazepam, zopiclone, or eszopiclone was permitted within protocol‐specified dose limits and timing constraints (not administered within 8 h prior to PANSS rating or other assessments).

### Efficacy assessments

The PANSS and CGI‐S were administered weekly throughout the study. The primary endpoint was change from baseline in PANSS total score at Week 6. Secondary endpoints included change from baseline in: PANSS total score at Weeks 1, 2, 3, 4, and 5, CGI‐S score at each post‐baseline visit, PANSS subscale scores at each post‐baseline visit, and PANSS 5‐factor Lindenmayer model^21^ scores, including negative symptoms, excitement, cognitive disorders, positive symptoms, and anxiety/depression, at each post‐baseline visit. Additional secondary outcomes included the proportion of subjects who achieved a clinical response, defined as 20% or greater improvement from baseline in PANSS total score at Week 6 using last observation carried forward (LOCF); change from baseline in Calgary Depression Scale for Schizophrenia (CDSS)^22^ total scores at Weeks 3 and 6; and time to all‐cause discontinuation from baseline. The EuroQOL‐5 Dimensions‐3 Levels (EQ‐5D‐3L)^23^ scale, evaluated by subjects using a paper scale at baseline and Week 6, was included as an exploratory endpoint.

### Safety assessments

Safety endpoints included assessment of treatment‐emergent adverse events (AE), laboratory tests, vital signs, waist circumference, bodyweight, body mass index, QTc interval determined from electrocardiography measurements, and use of concomitant antiparkinsonian drugs. The clinician‐rated Drug‐Induced Extrapyramidal Symptom Scale (DIEPSS)^24^ was used to assess extrapyramidal symptoms induced by antipsychotics. The emergence of suicidality was examined using the Columbia‐Suicide Severity Rating Scale (C‐SSRS).^25^ AE, vital signs, bodyweight, DIEPSS, and C‐SSRS were measured weekly throughout the 6‐week study and additionally at a Week‐7 follow‐up visit. Electrocardiography and laboratory tests were conducted at baseline and Week 6 (or end of study).

### Statistical analyses

The ITT population was defined as all randomized patients who received at least one dose of study drug and had both baseline and at least one post‐baseline assessment on the PANSS total score. The primary efficacy endpoint of change from baseline in the PANSS total score at Week 6 was analyzed using a mixed model for repeated measures (MMRM), with fixed factors of pooled study center, visit, treatment, and treatment‐by‐visit interaction, and baseline PANSS total score as a covariate. An unstructured covariance matrix was used for the within‐subject correlation and the Kenward‐Roger's approximation was used to calculate the denominator degree of freedom. The continuous secondary efficacy variables were analyzed in a similar way as done for the primary efficacy variable. Effect sizes for continuous measures on the primary and secondary variables were calculated as the absolute least squares mean difference divided by the model estimate of standard deviation. The counts, percentages, and odds ratio (OR) of PANSS responders at Week 6 were analyzed using a logistic regression model with terms for baseline PANSS total score, pooled study center, and treatment. Numbers needed to treat (NNT) were calculated. Time to all‐cause discontinuation was examined using a Kaplan–Meier product‐limit survival curve. Analyses of efficacy within the subgroup of Japanese patients focused on reporting of effect sizes comparing drug and placebo in change from baseline to Week 6 because the study was not powered for subgroup analyses. Safety was evaluated in the safety population, defined as all patients randomized who received at least one dose of study drug. All safety variables were summarized using descriptive statistics. For patients with missing data, PANSS responders are analyzed using LOCF method. All statistical inference, unless otherwise stated, was performed with two‐sided tests at the significance level of 0.05. All data analyses were conducted using SAS Version 9.4.

### Determination of sample size

In a previous clinical trial^14^ designed to evaluate the efficacy and safety of lurasidone (40 mg/day and 80 mg/day) treatment for 6 weeks compared to placebo in Asian (Japanese, Korean, Taiwanese, and Malaysian) subjects with acute schizophrenia, the effect size in change from baseline of the PANSS total score for the lurasidone group over placebo at Week 6 in the ITT population was 0.30 in the full sample and 0.37 in a subgroup of patients who met the inclusion/exclusion criteria of the present study. The effect size in the present study was estimated at 0.32, resulting in a sample size of 207 per treatment group to yield a 90% power with a two‐sided 5% significance level. Allowing for attrition, an inflation factor of 1.14 was calculated for an MMRM method as primary efficacy analysis using the method by Lu *et al*.,^26^ producing the target number of randomized subjects of 472 (236 per treatment group). Retention rates and correlation matrix used for the power calculation were estimated from the previous trial.

## Results

### Patient disposition and baseline characteristics

**Fig. 1:**
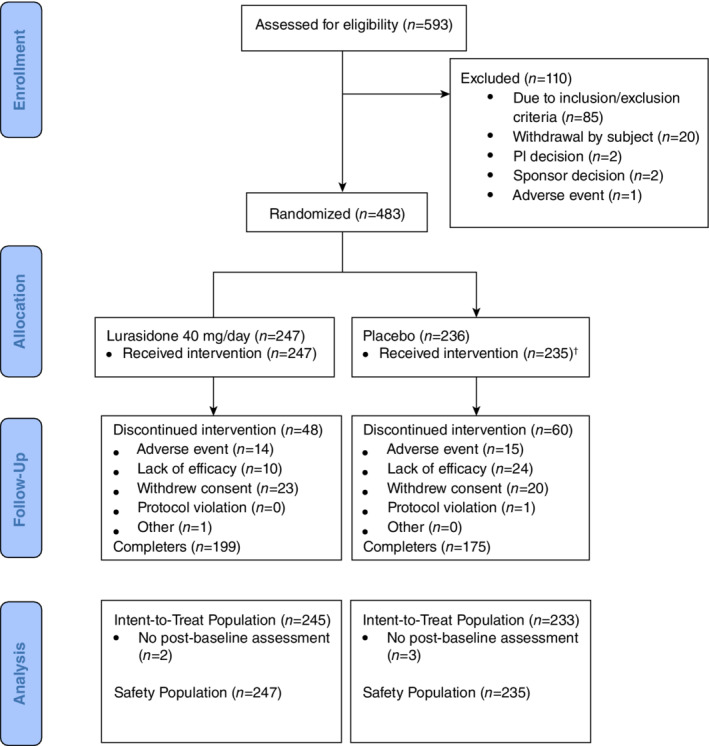
Patient disposition. †One subject did not receive study drug due to an important protocol deviation in the placebo group and was excluded from the intent‐to‐treat (ITT) and safety population. PI, principal investigator.

A total of 593 patients were screened for the study to achieve 483 randomized (Fig. 1). There were 247 randomized to the lurasidone group, all of whom received at least one dose of medication, and 236 randomized to placebo, one of whom did not receive the study drug and was excluded from the ITT and safety population. Two subjects in the lurasidone group and three in the placebo group had no post‐baseline PANSS assessments, leaving final ITT sample sizes of 245 and 233, respectively.

There were no subjects who were considered noncompliant. The most common reasons for discontinuing the intervention were withdrawal of consent, lack of efficacy, and adverse events. Demographic and clinical characteristics of the randomized sample were similar between the lurasidone and placebo groups (Table 1; Supplementary Materials Table S1). Sample sizes by country were Japan 107, Ukraine 193, Russia 146, Romania 23, and Poland 9.

**Table 1:** Baseline characteristics (ITT population)

	Placebo *N* = 233	Lurasidone *N* = 245	Overall *N* = 478
Sex, (Male), *n* (%)	120 (51.5)	119 (48.6)	239 (50.0)
Age (years), mean ± SD	39.3 ± 11.4	41.0 ± 11.0	40.2 ± 11.2
Race, *n* (%)			
White	183 (78.5)	183 (74.7)	366 (76.6)
Asian	50 (21.5)	59 (24.1)	109 (22.8)
Other	0	3 (1.2)	3 (0.6)
Country (Japan), *n* (%)	49 (21.0)	58 (23.7)	107 (22.4)
Duration of illness^†^ (years), mean ± SD	10.4 ± 8.3	10.6 ± 8.0	10.5 ± 8.2
Duration of current acute exacerbation of symptoms^‡^ (days), mean ± SD	20.0 ± 12.9	20.1 ± 13.1	20.1 ± 13.0
Number of prior hospitalizations (4 or more), *n* (%)	135 (57.9)	141 (57.6)	276 (57.7)
Diagnosis of paranoid type, *n* (%)	212 (91.0)	229 (93.5)	441 (92.3)
Body mass index (kg/m^2^), mean ± SD	25.2 ± 4.9	25.3 ± 4.5	25.2 ± 4.7
PANSS total score, mean ± SD	101.7 ± 11.5	102.8 ± 11.0	102.3 ± 11.3
PANSS positive subscale, mean ± SD	25.6 ± 3.9	25.9 ± 4.0	25.7 ± 3.9
PANSS negative subscale, mean ± SD	24.3 ± 4.4	24.6 ± 4.0	24.5 ± 4.2
PANSS general subscale, mean ± SD	52.3 ± 6.4	51.8 ± 6.6	52.0 ± 6.5^§^
CGI‐S score, mean ± SD	4.9 ± 0.6	5.0 ± 0.6	5.0 ± 0.6
Prior antipsychotic use, *n* (%)			
Typical	61 (26.2)	68 (27.8)	129 (27.0)
Atypical	106 (45.5)	116 (47.3)	222 (46.4)
Typical + atypical	56 (24.0)	48 (19.6)	104 (21.8)
Not used	10 (4.3)	13 (5.3)	23 (4.8)

Note: the decision to analyze the prior antipsychotic use was made after unblinding of the data.

^†^ Duration of illness indicates years from the initial episode of schizophrenia to the informed consent.

^‡^ Duration of current acute exacerbation of symptoms is days from the onset of current episode to the informed consent.

^§^ The mean ± SD was not pre‐specified by statistical analysis plan.

CGI‐S, Clinical Global Impressions‐Severity of Illness; PANSS, Positive and Negative Syndrome Scale; SD, standard deviation.

Concomitant medications in safety population were used by 42.1% (*n* = 104) of those in the lurasidone group and 45.1% (*n* = 106) of those in the placebo group. These were primarily anxiolytics (*n* = 66, 26.7%, for lurasidone; *n* = 74, 31.5% for placebo), most commonly lorazepam, and hypnotics/sedatives (*n* = 50, 20.2% for lurasidone; *n* = 53, 22.6% for placebo), most commonly brotizolam. Antiparkinsonian medications were used by 4.9% (*n* = 12) of those in the lurasidone group and 1.3% (*n* = 3) of those in the placebo group.

### Efficacy

**Fig. 2:**
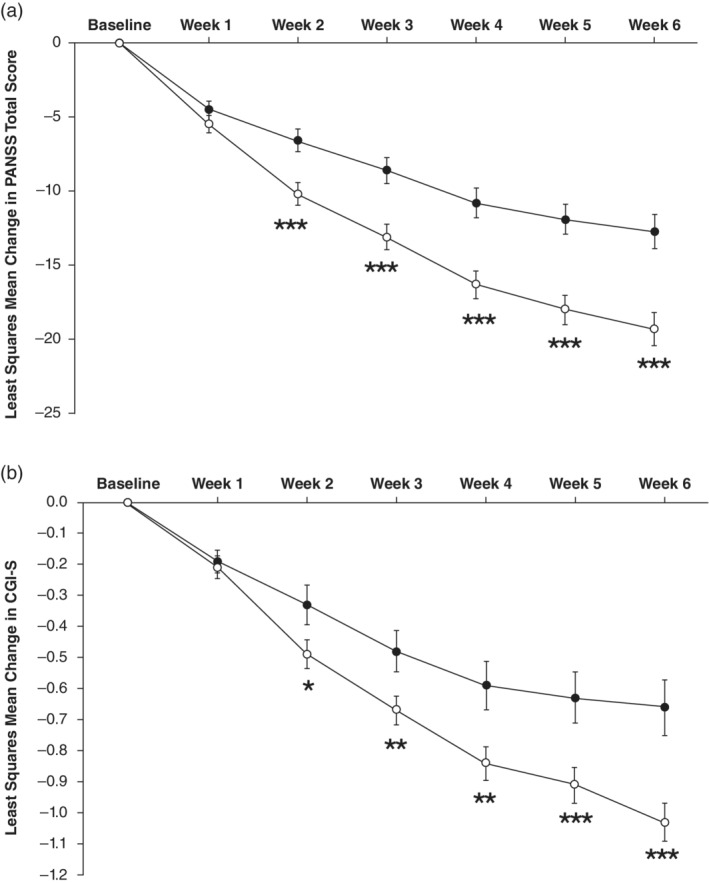
Change from baseline in (a) Positive and Negative Syndrome Scale (PANSS) total score, (b) Clinical Global Impression‐Severity Scale (CGI‐S) score in the intent‐to‐treat (ITT) population (*n* = 478). (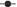) Placebo. (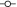) Lurasidone. *P < 0.05, **P < 0.01, ***P < 0.001 vs placebo. Data represent least squares mean estimate ± standard errors. MMRM, mixed model for repeated measures.

Using the MMRM model, the mean change from baseline to Week 6 in PANSS total score (primary endpoint) was significantly greater for the lurasidone group compared to the placebo group with a difference of −6.6 (standard error [SE] =1.58; 95% confidence interval [CI], −9.7, −3.5; *P* < 0.001; effect size = 0.41; LS mean of −19.3, −12.7, respectively). Treatment differences in change from mean baseline PANSS total score were observed at Week 2 and at subsequent visits (all had nominal *P*‐values < 0.001, not adjusted for multiple comparisons; Fig. 2a). Compared with placebo, treatment with lurasidone resulted in significantly greater proportions of PANSS responders at the LOCF endpoint (odds ratio [OR] = 2.36; 95% CI, 1.57, 3.55; NNT = 6). The response for lurasidone‐treated patients was 60.0% compared to 42.5% for placebo patients (Supplementary Materials Table S2).

Similar results were evident on the primary endpoint for the subgroup of patients in the Japan population. The reduction in mean PANSS total score from baseline to Week 6 was −13.7 for the lurasidone group and −6.8 for the placebo group for the treatment difference (SE) of −6.8 (3.52) (*P* = 0.054, effect size = 0.42). A treatment‐by‐country interaction included in the full ITT analysis was not significant (*P* = 0.836).

Treatment with lurasidone resulted in significantly greater reductions from mean baseline CGI‐S scores beginning at Week 2 (Fig. 2b). The effect size for the ITT population at Week 6 was 0.41 (*P* < 0.001 from MMRM analysis). Within the subgroup of patients from Japan, the reduction in mean CGI‐S scores from baseline to Week 6 was −0.7 for the lurasidone group and −0.6 for the placebo group (effect size = 0.08).

Compared to placebo‐treated patients, lurasidone‐treated patients showed significantly greater reductions from baseline to Week 6 on the PANSS Positive Symptom scale, PANSS Negative Symptom scale, PANSS General Psychopathology scale, and all of the PANSS Lindenmayer model scores (negative symptoms, excitement, cognitive disorders, positive symptoms, and anxiety/depression; Table 2). Effect sizes ranged from 0.22 to 0.44 across these measures. Within the subgroup of Japanese patients, effect sizes on these PANSS scales were similar, ranging from 0.21 to 0.48. All treatment‐by‐country (Japan vs others) interactions were not significant. The CDSS and the EQ‐5D‐3L index score did not show a significant difference in the full ITT population between lurasidone and placebo (Table 2 and Supplementary Tables S3a,b).

**Table 2:** Mean (SE) change from baseline to Week 6 on outcomes

	Full intent‐to‐treat sample				Japanese subgroup
	Placebo *N* = 233	Lurasidone *N* = 245	Effect size	*P‐*value	Effect size
PANSS total score	−12.7 (1.15)	−19.3 (1.10)	0.41	<0.001	0.42
CGI‐S score	−0.66 (0.07)	−1.03 (0.06)	0.41	<0.001	0.08
PANSS subscales					
Positive subscale	−3.9 (0.37)	−6.1 (0.35)	0.44	<0.001	0.35
Negative subscale	−2.5 (0.27)	−3.3 (0.26)	0.22	0.030	0.40
General Psychopathology subscale	−6.8 (0.61)	−10.0 (0.58)	0.39	<0.001	0.33
PANSS Lindenmayer 5‐factor scales					
Negative symptoms	−2.6 (0.27)	−3.4 (0.26)	0.23	0.019	0.33
Excitement	−1.5 (0.22)	−2.5 (0.21)	0.32	0.002	0.21
Cognitive disorders	−1.6 (0.18)	−2.4 (0.17)	0.31	0.002	0.48
Positive symptoms	−2.4 (0.23)	−3.7 (0.22)	0.40	<0.001	0.21
Anxiety/depression	−2.9 (0.23)	−4.0 (0.22)	0.34	<0.001	0.29
CDSS score	−1.17 (0.18)	−1.43 (0.17)	0.11	0.256	0.12
EQ‐5D‐3L index value	0.03 (0.01)	0.05 (0.01)	0.11	0.228	0.02^†^

^†^ The effect size was not pre‐specified by statistical analysis plan.

CDSS, Calgary Depression Scale for Schizophrenia; CGI‐S, Clinical Global Impressions‐Severity of Illness; EQ‐5D‐3L, EuroQOL‐5 Dimensions‐3 Levels; PANSS, Positive and Negative Syndrome Scale; SE, standard error.

The Kaplan–Meier plot for time to all‐cause discontinuation indicated that the 25th percentile was not reached for either lurasidone or placebo and median time to study treatment discontinuation could not be estimated.

### Safety

The incidence of any AE occurred in generally similar proportions between treatment groups (Table 3). Two patients in the lurasidone group had serious AE (schizophrenia; *n* = 2). Four patients in the placebo group had serious AE (schizophrenia, *n* = 2; hand fracture, *n* = 1; suicide attempt, *n* = 1). Fourteen patients in the lurasidone group and 15 in the placebo group had AE that led to study drug discontinuation. There were no deaths during the study.

**Table 3:** Summary of adverse events

	Placebo *N* = 235	Lurasidone *N* = 247
Overall		
Any AE	120 (51.1)	116 (47.0)
Serious AE	4 (1.7)	2 (0.8)
AE leading to discontinuation	15 (6.4)	14 (5.7)
AE of special interest		
Any extrapyramidal AE	12 (5.1)	20 (8.1)
Hyperglycemia/new‐onset diabetes	7 (3.0)	11 (4.5)
Weight gain	1 (0.4)	4 (1.6)
≥7% increase in weight	2 (0.9)	8 (3.3)
Hypersensitivity	8 (3.4)	11 (4.5)
Common adverse events^†^		
Akathisia	4 (1.7)	10 (4.0)
Dizziness	3 (1.3)	7 (2.8)
Somnolence	0	7 (2.8)
Abdominal discomfort	0	5 (2.0)
Asthenia	2 (0.9)	5 (2.0)

^†^ Treatment‐emergent adverse events reported in ≥2% on lurasidone and ≥ 2x placebo.

AE were classified using MedDRA Version 19.1.

Number (%) of patients is shown. AE, treatment‐emergent adverse event.

Common AE that occurred in more than 2% on lurasidone and at a rate at least twice that of the placebo group were akathisia (4.0%), dizziness (2.8%), somnolence (2.8%), abdominal discomfort (2.0%), and asthenia (2.0%; Table 3 and Supplementary Tables S4 and S5). There were 11 (4.5%) lurasidone‐treated patients who had a hyperglycemia/new onset diabetes AE compared to seven (3.0%) placebo‐treated patients. No patients had any dyslipidemia AE. Weight gain AE were reported by four (1.6%) patients in the lurasidone group and one (0.4%) patient in the placebo group. There were 11 (4.4%) patients in the lurasidone group and eight (3.4%) in the placebo group who had any hypersensitivity AE.

There were no clinically meaningful between‐group differences in the mean changes from baseline to Week 6 in blood chemistry values or serum prolactin (Table 4). No statistically significant between‐group differences were evident for change from baseline to Week 6 in weight gain, RR interval, PR interval, or QT interval (Table 4). No clinically meaningful changes in vital signs were observed from baseline to Week 6 in either treatment group.

**Table 4:** Baseline and mean change to Week 6 (LOCF) in weight, BMI, laboratory parameters, and ECG parameters (safety population)

Parameter mean (SD)	Placebo *N* = 235			Lurasidone *N* = 247		
	Baseline	Change	*n*	Baseline	Change	*n*
Bodyweight (kg)	71.90 (15.84)	−0.18 (1.96)	233	72.21 (14.78)	0.10 (2.07)	246
BMI (kg/m^2^)	25.24 (4.85)	−0.06 (0.70)	233	25.31 (4.48)	0.03 (0.74)	246
Waist circumference (cm)^†^	86.80 (13.00)	−0.26 (2.58)	226	86.35 (12.18)	−0.03 (3.31)	240
Triglycerides^‡^ (mg/dL)	127.07 (71.13)	−0.62 (61.59)	204	143.94 (136.58)	−14.33 (125.37)	211
Total cholesterol^‡^ (mg/dL)	183.86 (40.23)	−4.13 (32.99)	205	188.91 (48.27)	0.68 (56.75)	211
LDL cholesterol^‡^ (mg/dL)	107.10 (33.78)	−4.97 (26.39)	202	110.76 (41.88)	−1.83 (25.02)	208
HDL cholesterol^‡^ (md/dL)	51.84 (15.41)	0.91 (10.84)	204	51.31 (15.92)	0.79 (12.19)	211
Blood glucose^‡^ (mg/dL)	93.23 (12.28)	−0.08 (14.71)	205	92.92 (13.04)	0.44 (12.84)	211
Hemoglobin A1c (%)	5.25 (0.36)	−0.03 (0.23)	221	5.33 (0.37)	−0.03 (0.22)	232
Prolactin, overall (ng/mL)	17.57 (22.66)	−2.42 (22.93)	222	20.50 (30.90)	−1.59 (29.60)	234
Prolactin, men	10.85 (10.35)	−0.16 (10.46)	114	13.21 (11.69)	0.21 (12.40)	113
Prolactin, women	24.71 (29.14)	−4.81 (30.98)	108	27.40 (40.46)	−3.27 (39.39)	121
ECG: heart rate^†^(beats/min)	72.8 (12.8)	1.1 (12.7)	222	73.8 (12.2)	−1.6 (11.9)	240
ECG: RR interval^†^ (msec)	851.2 (153.6)	−10.2 (143.3)	222	837.2 (145.4)	18.5 (137.6)	240
ECG: PR interval^†^(msec)	151.0 (20.2)	−0.2 (13.7)	222	154.9 (21.9)	0.5 (14.8)	240
ECG: QTcF interval^†^(msec)	401.3 (19.1)	0.0 (16.9)	222	403.9 (19.8)	0.5 (14.9)	240

*P* < 0.05. Comparisons of the lurasidone vs placebo at LOCF endpoint are based on a rank ANCOVA analysis.

^†^ Significance testing was not pre‐specified by statistical analysis plan.

^‡^ Fasting was required per protocol.

BMI, body mass index; ECG, electrocardiogram; HDL, high‐density lipoprotein; LDL, low‐density lipoprotein.

At Week 6, adjusted LS means (SE) for DIEPSS total scores (excluded overall severity item) were similar between the lurasidone group (−0.16 [0.058]) and the placebo group (−0.14 [0.061]) (*P* = 0.817). The proportions of subjects who had at least one occurrence of suicidal ideation as measured by the C‐SSRS were similar between the lurasidone group (17 patients, 6.9%) and placebo group (16 patients, 6.8%). One patient in the lurasidone group and two patients in the placebo group attempted suicide.

## Discussion

In this 6‐week placebo‐controlled study, the efficacy and safety of lurasidone was demonstrated in acutely psychotic patients with schizophrenia recruited in Japan and other countries. Statistically significant differences between lurasidone and placebo on the PANSS total score were evident as early as Week 2 and were apparent at all subsequent visits, with a moderate effect size (0.41) at Week 6. The difference in improvements at endpoint was clinically meaningful as evidenced by a sizeable difference in response rates (60.0% for lurasidone; 42.5% for placebo) and single‐digit number needed to treat for response rate (NNT = 6). The effects of lurasidone were broad‐based: the CGI‐S and all subscale scores for the PANSS (Positive, Negative, and General Psychopathology) and PANSS 5‐factor Lindenmayer model (negative symptoms, excitement, cognitive disorders, positive symptoms, and anxiety/depression) scores showed significant superiority for lurasidone compared to placebo on change from baseline to Week 6.

The drug–placebo difference on the PANSS total score for the current study was similar to that in a previous trial for lurasidone in the treatment of schizophrenia. In that trial that recruited patients from the USA, Colombia, Lithuania, India, and the Philippines, Meltzer *et al*.^11^ found a slightly larger difference between lurasidone 40 mg/day and placebo in change from baseline to Week 6 on the PANSS total score (9.7 points difference). The baseline PANSS total score for the current study (mean = 102.3 ± 11.25), however, was approximately half a standard deviation higher than that in the Meltzer *et al*.^11^ trial (baseline mean PANSS total score of 97), indicating a somewhat more severe sample enrolled in the current study compared to the previous trial. In contrast, the Higuchi *et al*.^9^ trial that did not demonstrate a significant difference for lurasidone 40 mg, 80 mg or risperidone versus placebo had a much lower mean baseline PANSS total score (91.9 ± 16.8) that may have hindered finding a drug‐placebo difference. The low mean baseline severity of that trial was a function of a PANSS total score inclusion criterion set at ≥70, which was lower than the minimum PANSS severity that was required in the previous lurasidone trials.^10^, ^11^, ^12^ In addition, unlike the previous trials, the Higuchi *et al*.^9^ trial did not have a CGI‐S inclusion criterion and did not include patients with worsening psychotic symptoms at screening. Treatment‐resistant schizophrenia was also common in the sample of patients in the trial.^9^ Somewhat different inclusion/exclusion criteria were used in the second Higuchi *et al*.^14^ trial. In that trial, lurasidone 40 mg and 80 mg did not significantly separate from placebo on the primary outcome in a modified intent‐to‐treat analysis, but a full intent‐to‐treat analysis conducted as a secondary analysis did find that lurasidone 40 mg and 80 mg were superior to placebo. The PANSS total score indicated that severity of the sample at baseline was high (mean = 102.8 ± 15.5). However, the lack of drug‐placebo differences in this study may^14^ have been due to the enrollment of patients who had taken a total dose of antipsychotic medication equivalent to 12 mg or more haloperidol, and the high prevalence of both predominately negative symptoms and treatment‐resistant schizophrenia at screening. The inclusion and exclusion criteria of the current study were different from these trials (see Supplementary Tables S6a,b). The criteria of the current study included PANSS total score ≥ 80, CGI‐S score ≥ 4, inclusion of patients with acute schizophrenia, predominantly positive symptoms prior to screening, and exclusion of haloperidol‐equivalent dose ≥12 mg (to exclude patients with treatment‐resistant schizophrenia). These differences in inclusion criteria may explain the positive findings of the current study compared to the lack of drug‐placebo differences in the primary analyses of two previous trials conducted in Asian countries.^9^, ^14^

Because of the inconsistent results across the lurasidone trials including Japanese for acute schizophrenia, a network meta‐analysis was performed that included two previous trials and the current study.^27^ In this analysis, lurasidone 40 mg/day and 80 mg/day led to significant benefit in the treatment of acute schizophrenia. The results of these RCTs may have been inconsistent due to different selection criteria, but conducting such a network meta‐analysis suggests that lurasidone 40 mg/day is effective in patients with acute schizophrenia, including Japanese.

The pattern of adverse events found in the current study was similar to the AE profile observed in previous placebo‐controlled trials that evaluated lurasidone 40 mg/day in the treatment of schizophrenia. The consistent minimal effects on weight and metabolic parameters observed in this and previous studies have been attributed to the lack of clinically relevant affinity of lurasidone for the receptors (H_1_‐histamine and 5‐HT_2C_) that are thought be associated with weight gain.^7^, ^28^ The minimal effect of lurasidone on metabolic parameters is especially important given the high risk for metabolic syndrome that has been found for individuals with schizophrenia.^29^

Within the subgroup of Japanese patients, the treatment effect size (0.42) comparing lurasidone with placebo on change from baseline to Week 6 on the PANSS total score was nearly identical to that found in the overall sample (effect size = 0.41). Effect sizes for the PANSS subscales and Lindenmayer scores in the Japanese subgroup were also similar to those found in the overall sample; and all treatment‐by‐subgroup (Japan vs others) terms were non‐significant for primary and secondary measures. This suggests that lurasidone has similar efficacy for Japanese patients as it does for patients from other countries. This result, however, would need to be confirmed in a randomized, placebo‐controlled trial that is fully powered to detect differences within a Japanese sample.

The findings of this study should be understood in the context of some limitations. First, the short‐term (6‐week) nature of the study leaves open the question of longer‐term efficacy and safety. However, long‐term safety/tolerability has been verified in previous long‐term studies, including two 12‐month studies,^30^, ^31^ and one 28‐week double‐blind maintenance study^32^ in USA. Second, the study was conducted using a sample of individuals with acute symptoms of schizophrenia but excluding individuals continuously hospitalized for >14 days for acute exacerbation of psychotic symptoms immediately prior to screening and who had been treated continuously with adequate doses of one or more antipsychotic agents for ≥4 weeks immediately prior to screening (as well as other exclusion criteria).

In summary, lurasidone 40 mg once daily dosing demonstrated efficacy in a patient population, including subjects from Japan, with acute schizophrenia and predominantly positive symptoms; there seems to be no significant difference in ethnicity or nationality, similar to the results of previous US trials. It was generally safe and well‐tolerated.

## Disclosure statement

Dr. Iyo reports personal fees from Otsuka Pharmaceutical Co., Ltd., Jansen Pharmaceutical K.K, Kyowa Pharmaceutical Industry Co., Ltd., and Eli Lilly Japan, and also reports grants and personal fees from Sumitomo Dainippon Pharma and Takeda Pharmaceutical Co., personal fees and other from MSD K.K. Dr. Ishigooka reports personal fees from Novartis, Otsuka Pharmaceutical Co., Ltd., Sumitomo Dainippon Pharma, Eli Lilly Japan, Takeda Pharmaceutical Co, Alfresa Pharma, Lundbeck, and Yoshitomiyakuhin. Dr. Higuchi reports personal fees from Meiji Seika Pharma, MSD, Allergan, Eisai, Pfizer, Janssen, Lundbeck, Shionogi, Yoshitomi, Kyowa Pharmaceutical Industry, Mochida, Otsuka, Sumitomo Dainippon, Mitsubishi Tanabe, Eli Lilly, and Takeda. Nakamura, Sakaguchi, Okamoto, and Nosaka are full‐time employees of Sumitomo Dainippon Pharma. Mao, Tsai, and Fitzgerald are full‐time employees of Sunovion Pharmaceuticals.

## Author contributions

M.I, J.I and T.H: contributed to the conception and design of the study, acquisition and interpretation of results. M.N and Y.M: contributed to statistical data analysis of the study. R.S and J.T: contributed to conception and design of the study. K.O and A.F: contributed to acquisition of data and resource management of the study. T.N: contributed to conception and drafting of this manuscript. All authors contributed to and have approved the final manuscript.

## Supporting information

**Table S1.** Other baseline characteristics (intention‐to‐treat population).
**Table S2.** Proportion of PANSS Responders at Week 6 (last observation carried forward), logistic regression (intention‐to‐treat population).
**Table S3a.** Mean (standard error) change from baseline to Week 6 on EuroQOL‐5 Dimensions‐3 Levels index score. Analysis of covariance of change from baseline at Week 6 (intention‐to‐treat population).
**Table S3b.** EuroQOL‐5 Dimensions‐3 Levels Dimensions. Categorical summary (intention‐to‐treat population).
**Table S4.** Treatment‐emergent adverse events – Observed in ≥2.0% in lurasidone group (safety population).
**Table S5.** Treatment‐emergent adverse events leading to study drug discontinuation (safety population).
**Table S6a.** Inclusion criteria for each clinical trial.
**Table S6b.** Exclusion criteria for each clinical trial.

## References

[1] World Health Organization . *Schizophrenia Fact Sheet*. 2019. Available from URL: https://www.who.int/news-room/fact-sheets/detail/schizophrenia. Accessed on January 30, 2020.

[2] Global Burden of Disease Study 2013 Collaborators . Global, regional, and national incidence, prevalence, and years lived with disability for 301 acute and chronic diseases and injuries in 188 countries, 1990–2013: A systematic analysis for the Global Burden of Disease Study 2013. Lancet 2015; 386: 743–800. 26063472 10.1016/S0140-6736(15)60692-4 PMC4561509

[3] Harvey PD, Robert KH, Carpenter WT, Green MF, Gold JM, Schoenbaum M. Functional impairment in people with schizophrenia: Focus on employability and eligibility for disability compensation. Schizophr. Res. 2012; 140: 1–8. 22503642 10.1016/j.schres.2012.03.025 PMC3399960

[4] Correll CU, Solmi M, Veronese N*et al*. Prevalence, incidence and mortality from cardiovascular disease in patients with pooled and specific severe mental illness: A large‐scale meta‐analysis of 3,211,768 patients and 113,383,368 controls. World Psychiatry 2017; 16: 163–180. 28498599 10.1002/wps.20420 PMC5428179

[5] Hjorthoj C, Sturup AE, McGrath JJ, Nordentoft M. Years of potential life lost and life expectancy in schizophrenia: A systematic review and meta‐analysis. Lancet Psychiatry 2017; 4: 295–301. 28237639 10.1016/S2215-0366(17)30078-0

[6] Mitchell AJ, Vancampfort D, Sweers K, van Winkel R, Yu W, De Hert M. Prevalence of metabolic syndrome and metabolic abnormalities in schizophrenia and related disorders—a systematic review and meta‐analysis. Schizophr. Bull. 2013; 39: 306–318. 22207632 10.1093/schbul/sbr148 PMC3576174

[7] Ishibashi T, Horisawa T, Tokuda K*et al*. Pharmacological profile of lurasidone, a novel antipsychotic agent with potent 5‐hydroxytryptamine 7 (5‐HT7) and 5‐HT1A receptor activity. J. Pharmacol. Exp. Ther. 2010; 334: 171–181. 20404009 10.1124/jpet.110.167346

[8] Zheng W, Cai DB, Yang XH*et al*. Short‐term efficacy and tolerability of lurasidone in the treatment of acute schizophrenia: A meta‐analysis of randomized controlled trials. J. Psychiatr. Res. 2018; 103: 244–251. 29906709 10.1016/j.jpsychires.2018.06.005

[9] Higuchi T, Iyo M, Kwon JS*et al*. Randomized, double‐blind, placebo, and risperidone‐controlled study of lurasidone in the treatment of schizophrenia: Results of an inconclusive 6‐week trial. Asia Pacific Psychiatry 2019; 11: e12354. 30912222 10.1111/appy.12354

[10] Loebel A, Cucchiaro J, Sarma K*et al*. Efficacy and safety of lurasidone 80 mg/day and 160 mg/day in the treatment of schizophrenia: A randomized, double‐blind, placebo‐ and active‐controlled trial. Schizophr. Res. 2013; 145: 101–109. 23415311 10.1016/j.schres.2013.01.009

[11] Meltzer H, Cucchiaro J, Silva R*et al*. Lurasidone in the treatment of schizophrenia: A randomized, double‐blind, placebo‐ and olanzapine‐controlled study. Am. J. Psychiatry 2011; 168: 957–967. 21676992 10.1176/appi.ajp.2011.10060907

[12] Nasrallah H, Silva R, Phillips D*et al*. Lurasidone for the treatment of acutely psychotic patients with schizophrenia: A 6‐week, randomized, placebo‐controlled study. J. Psychiatr. Res. 2013; 47: 670–677. 23421963 10.1016/j.jpsychires.2013.01.020

[13] Kay S, Fiszbein A, Opler L. The Positive and Negative Syndrome Scale (PANSS) for schizophrenia. Schizophr. Bull. 1987; 13: 261–276. 3616518 10.1093/schbul/13.2.261

[14] Higuchi T, Ishigooka J, Iyo M*et al*. Lurasidone in the treatment of schizophrenia: Results of a double‐blind, placebo‐controlled trial in Asian patients. Asia Pacific Psychiatry 2019; 11: e12352. 30950208 10.1111/appy.12352

[15] Frackiewicz EJ, Sramek JJ, Herrera JM, Kurtz NM, Cutler NR. Ethnicity and antipsychotic response. Ann. Pharmacother. 1997; 31: 1360–1369. 9391692 10.1177/106002809703101114

[16] Lv D, Zhao M, Chen L*et al*. An inter‐ethnic comparison study of ziprasidone plasma levels, dosage and clinical response in patients with schizophrenia. Psychiatry Investig. 2017; 14: 360–367. 10.4306/pi.2017.14.3.360 PMC5440439 28539955

[17] Emsley RA, Roberts MC, Rataemane S*et al*. Ethnicity and treatment response in schizophrenia: A comparison of 3 ethnic groups. J. Clin. Psychiatry 2002; 63: 9–14. 10.4088/jcp.v63n0103 11841075

[18] Sheehan DV, Lecrubier Y, Sheehan KH*et al*. The Mini‐International Neuropsychiatric Interview (M.I.N.I.): The development and validation of a structured diagnostic psychiatric interview for DSM‐IV and ICD‐10. J. Clin. Psychiatry 1998; 59: 22–33. 9881538

[19] American Psychiatric Association . Diagnostic and Statistical Manual of Mental Disorders, 4th edn. American Psychiatric Press, Washington, DC, 2000.

[20] Guy W. ECDEU Assessment Manual for Psychopharmacology (Revised). National Institute of Mental Health, Rockville, MD, 1976.

[21] Lindenmayer JP, Bernstein‐Hyman R, Grochowski S. A new five factor model of schizophrenia. Psychiatry Q. 1994; 65: 299–322. 10.1007/BF02354306 7831416

[22] Addington D, Addington J, Maticka‐Tyndale E. Assessing depression in schizophrenia: The Calgary Depression Scale. Br. J. Psychiatry 1993; 163: 39–44. 8110442

[23] Brooks R, the EuroQol Group . EuroQol: The current state of play. Health Policy 1996; 37: 53–72. 10158943 10.1016/0168-8510(96)00822-6

[24] Inada T, Beasley C, Tanaka Y, Walker D. Extrapyramidal symptom profiles assessed with the Drug‐Induced Extrapyramidal Symptom Scale: Comparison with Western scales in the clinical double‐blind studies of schizophrenic patients treated with either olanzapine or haloperidol. Int. Clin. Psychopharmacol. 2003; 18: 39–48. 12490774 10.1097/00004850-200301000-00007

[25] Posner K, Brown GK, Stanley B*et al*. The Columbia‐Suicide Severity Rating Scale: Initial validity and internal consistency findings from three multisite studies with adolescents and adults. Am. J. Psychiatry 2011; 168: 1266–1277. 22193671 10.1176/appi.ajp.2011.10111704 PMC3893686

[26] Lu K, Luo X, Chen PY. Sample size estimation for repeated measures analysis in randomized clinical trials with missing data. Int. J. Biostat. 2008; 4: 9. 10.2202/1557-4679.1098 22462117

[27] Kishi T, Nosaka T, Sakuma K, Okuya M, Iwata N. Efficacy, tolerability, and safety of lurasidone for acute schizophrenia: A systematic review and network meta‐analysis of phase 3 trials in Japan. Neuropsychopharmacol. Rep. 2020; 40: 314–322. 32767739 10.1002/npr2.12131 PMC7722667

[28] Kroeze WK, Hufeisen SJ, Popadak BA*et al*. H1‐histamine receptor affinity predicts short‐term weight gain for typical and atypical antipsychotic drugs. Neuropsychopharmacology 2003; 28: 519–526. 12629531 10.1038/sj.npp.1300027

[29] Vancampfort D, Stubbs B, Mitchell AJ. Risk of metabolic syndrome and its components in people with schizophrenia and related psychotic disorders, bipolar disorder and major depressive disorder: A systematic review and meta‐analysis. World Psychiatry 2015; 14: 339–347. 26407790 10.1002/wps.20252 PMC4592657

[30] Citrome L, Cucchiaro J, Sarmab K*et al*. Long‐term safety and tolerability of lurasidone in schizophrenia: A 12‐month, double‐blind, active‐controlled study. Int. Clin. Psychopharmacol. 2012; 27: 165–176. 22395527 10.1097/YIC.0b013e32835281ef

[31] Loebel A, Cucchiaro J, Xu J, Sarma K, Pikalov A, Kane JM. Effectiveness of lurasidone vs quetiapine XR for relapse prevention in schizophrenia: A 12‐month, double‐blind, noninferiority study. Schizophr. Res. 2013; 147: 95–102. 23583011 10.1016/j.schres.2013.03.013

[32] Tandon R, Cucchiaro J, Phillips D*et al*. A double‐blind, placebo‐controlled, randomized withdrawal study of lurasidone for the maintenance of efficacy in patients with schizophrenia. J. Psychopharmacol. 2016; 30: 69–77. 26645209 10.1177/0269881115620460 PMC4717319

