# Supplementary material for: Efficacy and safety of lurasidone in acutely psychotic patients with schizophrenia: A 6‐week, randomized, double‐blind, placebo‐controlled study
Source: Psychiatry Clin Neurosci. 2021 May 21;75(7):227–35. doi: 10.1111/pcn.13221 (PMC8361730; doi:10.1111/pcn.13221)
Supplement: Supplementary file 1 — Table S1. Other baseline characteristics (intention‐to‐treat population). Table S2. Proportion of PANSS Responders at Week 6 (last observation carried forward), logistic regression (intention‐to‐treat population). Table S3a. Mean (standard error) change from baseline to Week 6 on EuroQOL‐5 Dimensions‐3 Levels index score. Analysis of covariance of change from baseline at Week 6 (intention‐to‐treat population). Table S3b. EuroQOL‐5 Dimensions‐3 Levels Dimensions. Categorical summary (intention‐to‐treat population). Table S4. Treatment‐emergent adverse events – Observed in ≥2.0% in lurasidone group (safety population). Table S5. Treatment‐emergent adverse events leading to study drug discontinuation (safety population). Table S6a. Inclusion criteria for each clinical trial. Table S6b. Exclusion criteria for each clinical trial. [file PCN-75-227-s001.docx]

**Supplementary Table S1**

Other baseline characteristics (ITT population).

|  | **Placebo**  ***N* = 233** | **Lurasidone**  ***N* = 245** | **Overall**  ***N* = 478** |
| --- | --- | --- | --- |
| **Duration of illness (years), *n* (%)** |  |  |  |
| **< 5** | 53 (22.7) | 55 (22.4) | 108 (22.6) |
| **≥ 5 to < 10** | 40 (17.2) | 33 (13.5) | 73 (15.3) |
| **≥ 10 to < 20** | 53 (22.7) | 56 (22.9) | 109 (22.8) |
| **≥ 20** | 20 (8.6) | 22 (9.0) | 42 (8.8) |
| **Number of prior hospitalizations, *n* (%)** |  |  |  |
| **0** | 22 (9.4) | 21 (8.6) | 43 (9.0) |
| **1** | 26 (11.2) | 35 (14.3) | 61 (12.8) |
| **2** | 28 (12.0) | 29 (11.8) | 57 (11.9) |
| **3** | 22 (9.4) | 19 (7.8) | 41 (8.6) |
| **4 or more** | 135 (57.9) | 141 (57.6) | 276 (57.7) |
| **PANSS Total Score, *n* (%)** |  |  |  |
| **< 101** | 116 (49.8) | 116 (47.3) | 232 (48.5) |
| **≥ 101** | 117 (50.2) | 129 (52.7) | 246 (51.5) |
| **PANSS Subscale Score, *n* (%)** |  |  |  |
| **Positive Subscale Score < Negative Subscale Score** | 89 (38.2) | 88 (35.9) | 177 (37.0) |
| **Positive Subscale Score ≥ Negative Subscale Score** | 144 (61.8) | 157 (64.1) | 301 (63.0) |
| **CDSS Score, mean ± SD** | 4.2 ± 3.8 | 4.2 ± 3.8 | 4.2 ± 3.8 |
| **EQ-5D-3L Index Score, mean ± SD** | 0.82 ± 0.17 | 0.79 ± 0.19 | 0.81 ± 0.18^†^ |

^†^ The mean ± SD was not pre-specified by statistical analysis plan.

CDSS=Calgary Depression Scale for Schizophrenia, EQ-5D-3L=Euroqol-5 dimensions-3 levels,

ITT=intention-to-treat, PANSS=Positive and Negative Syndrome Scale, SD=standard deviation.

**Supplementary Table S2**

Proportion of PANSS Responders at Week 6 (LOCF), Logistic Regression (ITT Population).

| **Improvement Level** | **Placebo**  **N = 233** | **Lurasidone**  **N=245** | **Comparison Between Treatment Groups**  **(Lurasidone versus Placebo)** | |
| --- | --- | --- | --- | --- |
|  | ***n* (%)** | ***n* (%)** | **Odds Ratio (95% CI)** | **NNT (95% CI)** |
| **≥ 20%** | 99 (42.5) | 147 (60.0) | 2.36 (1.57, 3.55) | 6 (3,12) |
| **≥ 30%** | 71 (30.5) | 108 (44.1) | 2.10 (1.36, 3.25) | 8 (4,20) |
| **≥ 40%** | 41 (17.6) | 58 (23.7) | 1.55 (0.94, 2.56) | 17 (-,-) |
| **≥ 50%** | 17 (7.3) | 30 (12.2) | 2.01 (0.99, 4.09) | 21 (-,-) |

CI = confidence interval, ITT=intention-to-treat, LOCF=last observation carried forward, n=number of subjects in the analysis with an event, NNT=numbers needed to treat, PANSS=Positive and Negative Syndrome Scale.

Response in PANSS total score at Week 6 (LOCF) was analyzed using a logistic regression model with terms for Baseline PANSS total score, pooled study center, and treatment. Proportion of subjects who achieved a PANSS response, defined as a 20% or greater improvement from Baseline in PANSS total score at Week 6 LOCF endpoint (additional definitions of response included 30%, 40%, and 50% or greater improvement from Baseline in PANSS total score). Baseline was defined as the last nonmissing measurement taken prior to or on the date of first dose of double-blind study medication. The LOCF endpoint was defined as the last post-Baseline assessment up to and including the Week 6 visit date, or up to and including 7 days following the date of last dose of double-blind study medication for subjects not entering the extension study. A lower CI and values for the odds ratio above 1.00 show lurasidone 40 mg to have a favorable outcome compared with placebo. The NNT was obtained as the reciprocal of the difference in the proportion of responders in the lurasidone group versus the placebo group. The 95% CI of NNT will be obtained by taking the reciprocal of the 95% CI bounds of the Absolute Risk Reduction when both lower and upper confidence limits are positive.

**Supplementary Table S3a**

Mean (SE) change from baseline to week 6 on EQ-5D-3L Index Score. −ANCOVA of change from Baseline at Week 6 (ITT Population).

|  | **Placebo**  ***N* = 233** | **Lurasidone**  ***N* = 245** | **Difference Between Treatment Groups** | |
| --- | --- | --- | --- | --- |
|  | **Adjusted LS**  **Mean (SE)**  ***n* = 222** | **Adjusted LS Mean (SE)**  ***n* = 239** | **Effect Size** | ***P* value** |
| **EQ-5D-3L Index Value** | 0.03 (0.010) | 0.05 (0.010) | 0.11 | 0.228 |
| **EQ VAS Score** | 4.93 (1.304) | 8.78 (1.257) | 0.20 | 0.030 |

ANCOVA=analysis of covariance, EQ-5D-3L=Euroqol-5 dimensions-3 levels, EQ VAS=Euroqol visual analogue scale, ITT=intention-to-treat, LS=least squares, *N* = number of subjects in the treatment group, *n*=number of subjects in the analysis, SE=standard error.

Change from Baseline in EQ-5D-3L value at Week 6 was analyzed using an ANCOVA model with treatment group as fixed effect and pooled study center and Baseline EQ-5D-3L value as covariates.

Note: the decision to analyze the EQ VAS score was made after unblinding of the data.

**Supplementary Table S3b**

EQ-5D-3L Dimensions – Categorical Summary (ITT Population).

| **Dimension of Health** | **Level** | **visit** | **Placebo**  ***N* = 233**  ***n* (%)** | **Lurasidone**  ***N* = 245**  ***n* (%)** |
| --- | --- | --- | --- | --- |
| **Mobility** | **No problems in walking** | **Baseline** | 203 (87.1) | 202 (82.4) |
|  |  | **Week 6** | 192 (82.4) | 209 (85.3) |
| **Self-Care** | **No problems with self-care** | **Baseline** | 183 (78.5) | 175 (71.4) |
|  |  | **Week 6** | 189 (81.1) | 201 (82.0) |
| **Usual Activities** | **No problems in performing my usual activities** | **Baseline** | 108 (46.4) | 101 (41.2) |
|  |  | **Week 6** | 125 (53.6) | 134 (54.7) |
| **Pain/Discomfort** | **No pain or discomfort** | **Baseline** | 150 (64.4) | 149 (60.8) |
|  |  | **Week 6** | 160 (68.7) | 163 (66.5) |
| **Anxiety/Depression** | **Not anxious or depressed** | **Baseline** | 82 (35.2) | 72 (29.4) |
|  |  | **Week 6** | 112 (48.1) | 141 (57.6) |

EQ-5D-3L = Euroqol-5 dimensions-3 levels. ITT=intention-to-treat.

Note: the decision to analyze the EQ-5D-3L items was made after unblinding of the data.

**Supplementary Table S4**

Treatment-emergent adverse events – Observed in ≥ 2.0% in lurasidone group (Safety Population).

|  | **Placebo**  ***N* = 235**  ***n* (%)** | **Lurasidone**  ***N* = 247**  ***n* (%)** |
| --- | --- | --- |
| **Headache** | 11 (4.7) | 15 (6.1) |
| **Insomnia** | 27 (11.5) | 15 (6.1) |
| **Schizophrenia** | 22 (9.4) | 15 (6.1) |
| **Akathisia** | 4 (1.7) | 10 (4.0) |
| **Nasopharyngitis** | 7 (3.0) | 9 (3.6) |
| **Anxiety** | 16 (6.8) | 8 (3.2) |
| **Dizziness** | 3 (1.3) | 7 (2.8) |
| **Somnolence** | 0 | 7 (2.8) |
| **Abdominal discomfort** | 0 | 5 (2.0) |
| **Asthenia** | 2 (0.9) | 5 (2.0) |
| **Constipation** | 4 (1.7) | 5 (2.0) |

*N* = Number of subjects in treatment group. *n* = Number of subjects in analysis.

Events are sorted by decreasing frequency of preferred term in the lurasidone column.

**Supplementary Table S5**

Treatment-emergent Adverse Events Leading to Study Drug Discontinuation (Safety Population).

|  | **Placebo**  ***N* = 235**  ***n* (%)** | **Lurasidone**  ***N* = 247**  ***n* (%)** |
| --- | --- | --- |
| **Schizophrenia** | 11 (4.7) | 7 (2.8) |
| **Anxiety** | 0 | 1 (0.4) |
| **Catatonia** | 0 | 1 (0.4) |
| **Delusion** | 0 | 1 (0.4) |
| **Psychotic disorder** | 1 (0.4) | 1 (0.4) |
| **Atrioventricular block** | 0 | 1 (0.4) |
| **Pulmonary tuberculosis** | 0 | 1 (0.4) |
| **Weight increased** | 0 | 1 (0.4) |
| **Hand fracture** | 1 (0.4) | 0 |
| **Hostility** | 1 (0.4) | 0 |
| **Suicide attempt** | 1 (0.4) | 0 |

*N* = Number of subjects in treatment group. *n* = Number of subjects in analysis.

Events are sorted by decreasing frequency of preferred term in the lurasidone column.

**Supplementary Table S6a**

Inclusion criteria for each clinical trial.

|  | **Mainly Japan Phase III study** | | | **Mainly US Phase III Study** | | |
| --- | --- | --- | --- | --- | --- | --- |
| **Study name** | **Higuchi 2019^9^** | **Higuchi 2019^14^** | **Current Study** | **Nasrallah 2013^12^** | **Meltzer 2011^11^** | **Loebel 2013^10^** |
| **Age** | ≥18 and <75 years | ≥18 and ≤74 years | | ≥18 and ≤75 years | | |
| **Diagnosis, schizophrenia subtype** | Diagnosed with schizophrenia by DSM-IV | Met DSM-IV-TR criteria for schizophrenia with disorganized, paranoid, and undifferentiated subtypes. | | Met DSM-IV criteria for schizophrenia with disorganized, paranoid, and undifferentiated subtypes. | | Met DSM-IV-TR criteria for schizophrenia with disorganized, paranoid, and undifferentiated subtypes. |
| **Duration of current**  **episode** | － | Had an exacerbation of psychotic symptoms within 60 days prior to the screening | Had an acute exacerbation of psychotic symptoms within 60 days prior to screening and had marked deterioration of function (by history) from baseline (before exacerbation), and presented with exacerbation of mainly positive symptoms (eg, exacerbation of delusion or hallucination, thought disorder). | Had an acute exacerbation of psychotic symptoms within 2 months) or had been hospitalized for the purpose of treating an acute psychotic exacerbation for 2 consecutive weeks or less immediately before screening. | | |
| **Duration of illness** | － | | | ≥1 year | | |
| **PANSS total score** | ≥70 | ≥80 | | | | |
| **PANSS subscale scores** | ≥4 on at least 1 item of positive subscale scores | ≥ 4 on 2 or more of the following 5 items:  delusions (P1)  conceptual disorganization (P2)  hallucinations (P3)  suspiciousness (P6)  unusual thought content (G9) | | | | ≥ 4 on 2 or more of the following 4 items:  delusions (P1)  conceptual disorganization (P2) hallucinations (P3)  unusual thought content (G9) |
| **CGI-S score** | － | ≥4 | | | | |

CGI-S=Clinical Global Impressions-Severity of Illness, DSM-IV(-TR)=Diagnostic and Statistical Manual of Mental Disorder, 4th Edition, (Text Revision), PANSS= Positive and Negative Syndrome Scale.

**Supplementary Table S6b.**

Exclusion criteria for each clinical trials.

|  | **Mainly Japan Phase III study** | | | **Mainly US Phase III Study** | | |
| --- | --- | --- | --- | --- | --- | --- |
| **Study name** | **Higuchi 2019^9^** | **Higuchi 2019^14^** | **Current Study** | **Nasrallah 2013^12^** | **Meltzer 2011^11^** | **Loebel 2013^10^** |
| **Treatment resistance** | Failure to respond to 3 or more antipsychotic agents within 1 year before screening | | Failure to respond to 2 or more antipsychotic agents, given at an adequate dose for at least 4 weeks (28 consecutive days) within 1 year before screening | Failure to respond to 2 or more antipsychotic agents of different types within 1 year before screening | Failure to respond to 2 or more antipsychotic agents of different types for at least 8 weeks within 1 year before screening | Failure to respond to 2 or more marketed antipsychotic agents from 2 different classes, given at an adequate dose for at least 6 weeks. |
|  | Receiving a total dose of antipsychotic medication equivalent to >12.0 mg/day of haloperidol at the screening visit | | Receiving a total dose of antipsychotic medication equivalent to ≥12.0 mg/day of haloperidol at the screening visit | － | | |
| **Hospitalization** | － | | Continuously hospitalized for >3 months (90 days) immediately prior to screening. | － | | |
| **Placebo**  **responder** | a decrease of ≥20% in PANSS total score between screening and baseline visits | | | | | |

PANSS= Positive and Negative Syndrome Scale.
